# Presenilin 1 Mutations Converge on Aβ42/Aβ40 Elevation via Distinct Mechanisms

**DOI:** 10.1002/mco2.70978

**Published:** 2026-09-30

**Authors:** Isabel Bestard‐Lorigados, Keenan Sterling, Liwei Li, Zhehao Lin, Zhanghui Wu, Qinxin Zhu, Wenhao Pan, Fang Cai, Weihong Song

**Affiliations:** ^1^ Department of Psychiatry Townsend Family Laboratories Graduate Program in Neuroscience The University of British Columbia Vancouver Canada; ^2^ Center For Geriatric Medicine Zhejiang Key Laboratory of Alzheimer's Disease and Institute of Aging The First Affiliated Hospital of Wenzhou Medical University Wenzhou China; ^3^ Oujiang Laboratory (Zhejiang Lab For Regenerative Medicine, Vision and Brain Health) Wenzhou China

**Keywords:** Alzheimer's disease, amyloid β peptides, BACE1, endoproteolysis, presenilin 1

## Abstract

Mutations in Presenilin 1 (PS1) are the most common genetic cause of familial Alzheimer's disease (AD). However, the exact mechanisms by which these mutations alter amyloid precursor protein (APP) processing and drive amyloid β (Aβ) production remain unclear. In this study, five PS1 mutations (M146V, G384A, C410Y, Y115H, and the Notch‐sparing mutation ΔS169) were transfected into neuroblastoma cells lacking endogenous PS1/PS2 expression and human embryonic kidney cells stably overexpressing either wild‐type APP or the APP Swedish mutation. We investigated the effects of these mutations on APP processing, Aβ42/Aβ40 ratio, cytotoxicity, BACE1 expression, and PS1 endoproteolysis. Our results demonstrate that PS1 mutations differentially modulate APP processing, producing distinct Aβ profiles independent of their effects on Notch signaling. All mutations increased the Aβ42/Aβ40 ratio, while BACE1 levels were unchanged and no direct PS1‐BACE1 interactions were found. All mutations increased cytotoxicity, whereas only the Notch‐impairing mutations reduced cell viability, suggesting impaired Notch signaling may contribute to this effect. Lastly, the mutations exhibited distinct endoproteolytic rates that correlated only with APP‐C83 levels. These findings provide novel insights into how distinct PS1 mutations impact amyloid pathology.

## Graphical abstract

Presenilin 1 (PS1) mutations are the leading genetic cause of familial Alzheimer's disease, yet their specific effects on amyloid pathology remain unclear. This paper provides novel insights into the effects of five pathological PS1 mutations on amyloid‐precursor protein (APP) processing, cellular health, and endoproteolysis. These mechanisms contribute to mutation‐specific pathways likely driving amyloid pathology in familial Alzheimer's disease. Figure created with BioRender.

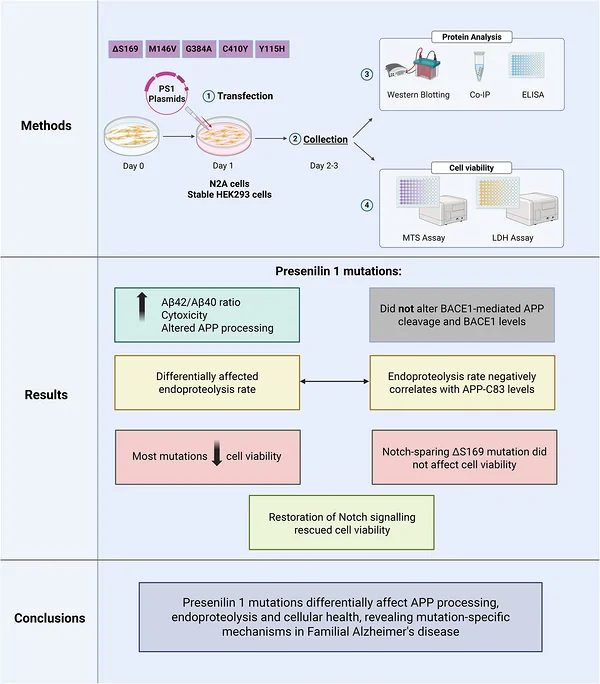

## 1 Introduction

Alzheimer's disease (AD) is the leading cause of dementia worldwide. A unique feature of AD is the formation of neuritic plaques via the accumulation of amyloid β peptides (Aβ) in the brain [1]. Aβ is generated through the sequential cleavage of the amyloid precursor protein (APP) by β‐ and γ‐secretases [2]. Under physiological conditions, APP is primarily cleaved by α‐secretase at the Leucine 17 (L17) site within the Aβ domain, producing soluble APPα (sAPPα) and an APP C‐terminal fragment (CTF) called C83, precluding Aβ generation [3, 4, 5]. Interestingly, under physiological conditions, beta‐site APP‐cleaving enzyme 1 (BACE1) also cleaves APP within the Aβ domain at the Glutamic acid 11 (E11) site, generating a distinct CTF called C89 that is further cleaved by γ‐secretase to produce truncated forms of Aβ [6, 7, 8]. In contrast, under pathological conditions associated with AD, BACE1 shifts its APP cleavage site to the Aspartic acid 1 (D1) site, resulting in the production of a shorter soluble APP fragment (sAPPβ) and a longer CTF called C99 [7, 8]. C99 is subsequently cleaved by γ‐secretase to create Aβ peptides [9].

Inhibiting γ‐secretase has been studied as a potential therapeutic target for AD due to its crucial role in the production of Aβ. However, previous clinical trials involving γ‐secretase inhibitors have failed because of severe side effects, primarily caused by the disruption of Notch processing [9, 10, 11, 12]. Importantly, mutations in presenilin 1 (PS1), the catalytic subunit of the γ‐secretase complex, are the most common genetic cause of familial AD (FAD) [13, 14]. Most PS1 mutations increase the Aβ42/Aβ40 ratio and disrupt Notch processing. Interestingly, Notch‐sparing FAD‐linked PS1 mutations, such as our recently characterized PS1_ΔS169_ [13], suggest that specific motifs within PS1 could be targeted to modulate APP cleavage selectively without impairing Notch signaling.

The different molecular mechanisms by which PS1 mutations that impair Notch signaling and the one that spares Notch operate, along with their effects on APP processing, BACE1 activity, and downstream signaling, are still unclear. Additionally, the PS1 protein itself undergoes a process of catalyzing its cleavage, called endoproteolysis [15, 16, 17], generating a 28 kDa N‐terminal fragment (NTF) and a 18 kDa CTF. However, it is unclear whether PS1 endoproteolysis is required for proper γ‐secretase activity, as the endoproteolysis‐deficient mutant PS1_ΔE9_ retains enzymatic function [18]. Furthermore, other research suggests that impairment of PS1 endoproteolysis can lead to differential APP processing and Aβ generation [19]. This raises the possibility that PS1 mutations could influence APP processing by altering PS1 stability. Studies in cellular and transgenic mouse models have shown that FAD‐causing PS1 mutations are associated with neurotoxicity and cell death [20, 21, 22, 23]. However, because the Notch signaling pathway also regulates cell fate and apoptosis [24, 25, 26], it remains unclear whether impaired Notch signaling contributes to the neurotoxic effects of these mutations.

PS1 mutations are distributed throughout the protein and are frequently located within transmembrane domains, highlighting the structural and functional diversity of these variants. In this project, five PS1 mutations (PS1_ΔS169_, PS1_Y115H_, PS1_M146V_, PS1_G384A_, and PS1_C410Y_) are examined, representing both Notch‐sparing and Notch‐impairing variants located across different domains of the PS1 protein. The ΔS169 mutation is an early‐onset FAD‐associated variant first reported in a family from China [27]. This mutation is a trinucleotide deletion that results in the loss of Serine 169, a residue critical for interactions among PS1, APP, and Notch [28, 29]. Previous work from our lab showed that PS1_ΔS169_ increases Aβ42/Aβ40 ratios and plaque burden without disrupting Notch processing [11, 13], making it uniquely valuable for studying substrate‐specific γ‐secretase function. The PS1_M146V_ mutation (methionine to valine at codon 146) is one of the most common FAD variants, with disease onset typically between the late 30s and 50s [30, 31]. It is associated with severe pathology, including increased Aβ42/Aβ40 ratios [32, 33], accelerated plaque formation, and cognitive decline [34, 35]. This mutation affects both APP and Notch processing [11, 13], making it a key model for widespread γ‐secretase dysfunction. The G384A mutation (glycine to alanine at residue 384) is located in transmembrane domain 7, near the catalytic residues within the conserved GxGD motif, a critical region for γ‐secretase activity [36]. Clinically, it is associated with aggressive disease progression and very early onset in the early 30s to 40s [37]. Its location and strong impact on both APP and Notch processing [33, 38, 39] make it very useful for studying substrate cleavage mechanisms. The C410Y mutation (cysteine to tyrosine at position 410) occurs in transmembrane domain 8 and is associated with symptom onset between 40 and 60 years of age [40]. This mutation alters γ‐secretase activity, affects APP processing, and disrupts Notch signaling [11, 13], leading to increased production of longer Aβ species [39, 41]. Finally, the Y115H mutation (tyrosine to histidine at position 115) is located in the first loop between transmembrane domains 1 and 2 and is linked to early‐onset AD (30s–40s) [42, 43]. It has been associated with increased Aβ42/Aβ40 ratios, reduced production of shorter Aβ species, and impaired Notch cleavage [13, 38, 44].

In this study, we demonstrate that these PS1 mutations alter APP processing in vitro, independent of their effects on Notch signaling. They also increase the Aβ42/Aβ40 ratio and differentially impact Aβ40 and Aβ42 levels. Consistent with previous reports, the mutations increased cytotoxicity; however, the Notch‐sparing mutation was the only one that did not reduce cell viability. Moreover, we show that PS1 does not directly interact with BACE1 in vitro, and that the mutations do not affect BACE1 levels or APP CTFβ production. Lastly, we found that specific PS1 mutations impair endoproteolytic processing to varying degrees, and this impairment correlated with aberrant cleavage of APP in vitro.

## 2 Results

### 2.1 PS1 Mutations Alter APP Processing and Elevate the Aβ42/Aβ40 Ratio Through Distinct Effects on Aβ Production

To investigate whether these FAD‐associated PS1 mutations modify APP processing under pathological conditions, 20E2 cells that stably expressed the APP_SWE_ mutation [45] were transfected with PS1_WT_ or mutant plasmids. Western blot analysis revealed that all mutations significantly increased C83 levels compared with PS1_WT_. Specifically, PS1_∆S169_ had a 2.29 ± 0.24 fold increase (*p* < 0.001), while PS1_M146V_, PS1_G384A_, PS1_C410Y_, and PS1_Y115H_ increased C83 levels by 1.76 ± 0.29 (*p* < 0.05), 1.98 ± 0.23 (*p* < 0.01), 1.76 ± 0.09 (*p* < 0.05), and 1.8 ± 0.2 (*p* < 0.05), respectively (Figure 1A,B). To further determine whether the observed changes in APP‐CTFs were due to altered APP expression rather than processing, APP full‐length (APP‐FL) levels were assessed. APP‐FL levels were not significantly different between any of the PS1 mutants and PS1_WT_ in 20E2 (*p* > 0.05) (Figure S1A,B), indicating that these PS1 mutations do not affect APP expression, but rather APP processing.

**FIGURE 1:**
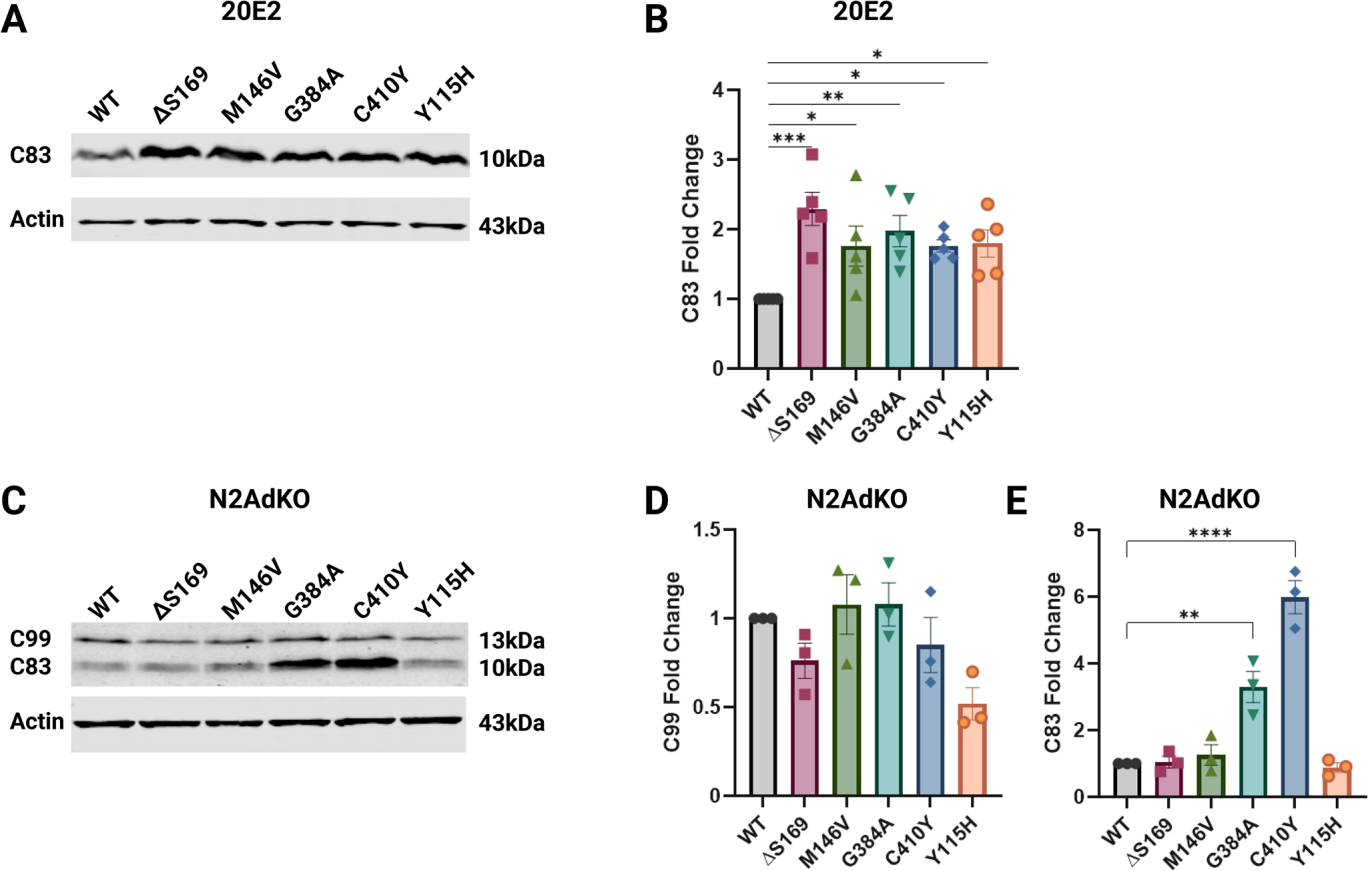
PS1 mutations alter APP processing by differentially affecting APP‐CTF levels in 20E2 and N2AdKO cells. (A) Representative Western blots showing C83 levels in 20E2 cells transfected with PS1_WT_ or mutant PS1 constructs: PS1_ΔS169_, PS1_M146V_, PS1_G384A_, PS1_C410Y_, and PS1_Y115H_, *n* = 5. (B) Quantification of C83 levels in 20E2 cells normalized to PS1_WT_. Rabbit anti‐APP antibody C20 detected C83 levels, while β–actin was detected by monoclonal mouse anti‐β‐actin antibody. All five mutations significantly increased C83 levels; *p < 0.05, **p < 0.01, ***p < 0.001. (C) Representative Western blots showing C99 and C83 levels in N2AdKO cells co‐transfected with C99 and previously mentioned PS1 variants, *n* = 3. (D) Quantification of C99 levels in N2AdKO cells, detected by the C20 antibody. No significant changes were observed between PS1_WT_ and mutants; *p* > 0.05. (E) Quantification of C83 levels in N2AdKO cells. PS1_G384A_ and PS1_C410Y_ significantly increased C83 levels compared with PS1_WT_; **p < 0.01, ****p < 0.0001. Approximate molecular weights are indicated: β‐actin (∼43 kDa), C99 (∼13 kDa), and C83 (∼10 kDa). Data are shown as mean ± SEM. Statistical analyses were determined by one‐way ANOVA with Dunnett's post hoc test.

To determine whether other proteins relevant to APP cleavage are affected by these mutations, PS2 and alpha secretase‐related proteins (ADAM10, ADAM17) levels were quantified in 20E2 cells after transfection of the PSI variants. Levels of the three proteins were not significantly different for any mutations compared with PS1_WT_ (*p* > 0.05) (Figure S2A–F).

To assess APP processing independently of endogenous presenilins in a neuronal cell model, mouse N2AdKO neuroblastoma cells (PS1/PS2 double knockout) were co‐transfected with PS1 plasmids and a C99 construct. C99 levels did not significantly differ between PS1_WT_ and any of the mutants (fold changes for PS1_∆S169_: 0.76 ± 0.1; PS1_M146V_: 1.08 ± 0.17; PS1_G384A_: 1.14 ± 0.12; PS1_C410Y_: 0.92 ± 0.15; PS1_Y115H_: 0.54 ± 0.09; all *p* > 0.05) (Figure 1C,D). However, C83 levels were significantly increased in cells expressing PS1_G384A_ and PS1_C410Y_ mutations (fold changes of 3.3 ± 0.47, *p* < 0.01 and 5.99 ± 0.5, *p* < 0.0001, respectively) (Figure 1C,E), whereas PS1_∆S169_, PS1_M146V_, and PS1_Y115H_ mutations had no significant effect (fold changes: 1.05 ± 0.17, 1.26 ± 0.31, and 0.88 ± 0.14; *p* > 0.05) compared with PS1_WT_.

To examine whether alpha‐secretase‐related proteins were affected in N2AdKO cells by PS1 variants, Western Blots were performed to measure ADAM10 and ADAM17 levels. Levels of these two proteins were not significantly different for any mutations compared with PS1_WT_ (*p* > 0.05) (Figure S3A–D).

To investigate how these mutations influence Aβ production, the extracellular levels of Aβ40 and Aβ42 were quantified via ELISA in N2AdKO cells co‐transfected with C99 and PS1 variants. Notably, compared with PS1_WT_ (concentration of 4024 ± 88.36), all mutations significantly decreased Aβ40 (*p* < 0.0001 for all) with concentrations of 2599 ± 47.96 for PS1_∆S169_, 2002 ± 63.52 for PS1_M146V_, 281.4 ± 2.37 for PS1_G384A_, 233.9 ± 1.52 for PS1_C410Y_, and 1594 ± 15.45 for PS1_Y115H_ (Figure 2A). Aβ42 levels were significantly increased (*p* < 0.0001) for PS1_∆S169_ (Aβ42 concentration of 942 ± 4.45), PS1_M146V_ (571.5 ± 4.65), and PS1_Y115H_ (666.8 ± 8.14) compared with PS1_WT_ (451.4 ± 8.37), while PS1_G384A_ (163.5 ± 1.25) and PS1_C410Y_ (56.51 ± 2.38) significantly decreased (*p* < 0.0001) Aβ42 levels (Figure 2B) compared with PS1_WT_. Consequently, all mutations increased the Aβ42/Aβ40 ratio significantly (*p* < 0.0001 for all), with PS1_WT_ at an Aβ42/Aβ40 concentration of 0.1122 ± 0.0012 and mutant values of 0.3627 ± 0.0074 for PS1_∆S169_, 0.2862 ± 0.0117 for PS1_M146V_, 0.581 ± 0.007 for PS1_G384A_, 0.2415 ± 0.0093 for PS1_C410Y_, and 0.4184 ± 0.0198 for PS1_Y115H_ (Figure 2C). Combined, these results show that PS1 mutations can affect APP processing, significantly increasing the ratio of Aβ42 to Aβ40, while having distinct effects on Aβ40 and Aβ42 concentrations.

**FIGURE 2:**
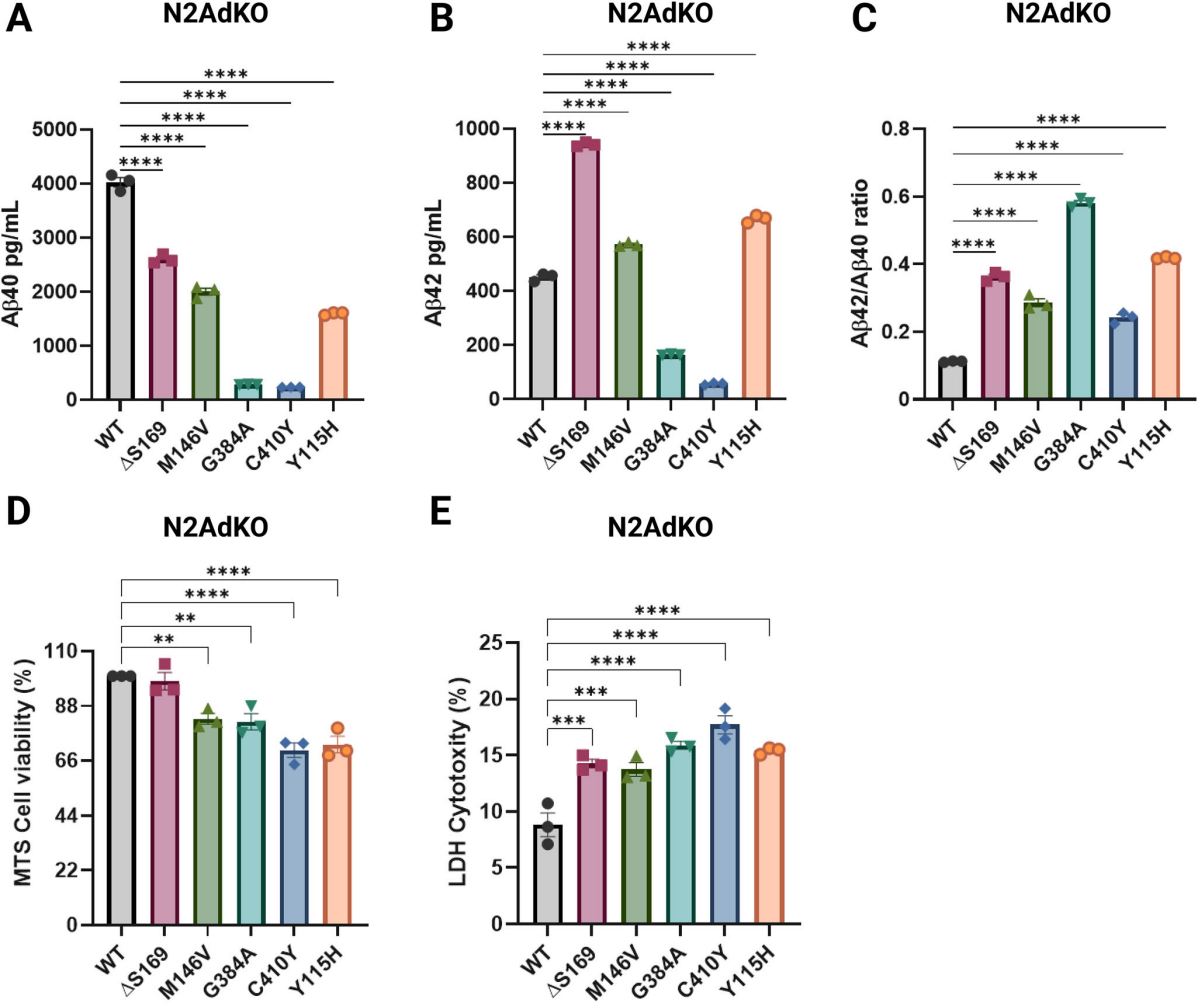
PS1 mutations alter Aβ production and increase cytotoxicity in N2AdKO cells. N2AdKO cells were transfected with PS1 variants, including PS1_WT_, PS1_ΔS169_, PS1_M146V_, PS1_G384A_, PS1_C410Y_, and PS1_Y115H_. For Aβ measurements, cells were co‐transfected with C99 and PS1 variants, and conditioned media were collected for ELISA; *n* = 3. (A) ELISA quantification of extracellular Aβ40 levels. All PS1 mutants showed a significant decrease in Aβ40 compared with PS1_WT_; ****p < 0.0001. (B) ELISA quantification of extracellular Aβ42 levels. PS1_∆S169_, PS1_M146V_, and PS1_Y115H_ increased Aβ42 levels, while PS1_G384A_ and PS1_C410Y_ decreased them; ****p < 0.0001. (C) Aβ42/Aβ40 ratios were significantly elevated for all PS1 mutants compared with PS1_WT_ (****p < 0.0001). (D) MTS assay results showing relative cell viability percentage normalized to PS1_WT_. All mutations except PS1_ΔS169_ (*p* > 0.05) significantly reduced viability; **p < 0.01, ****p < 0.0001, *n* = 3. (E) LDH cytotoxicity assay results showing increased LDH release as a percentage of cytotoxicity for all PS1 mutants compared with PS1_WT_; ***p < 0.001, ****p < 0.0001; *n* = 3. Data are shown as mean ± SEM. Statistical analyses were conducted by one‐way ANOVA with Dunnett's post hoc test.

### 2.2 PS1 Mutations Increase Cytotoxicity, While Cell Viability Impairments Are Mutation‐Specific and Rescued by Notch Signaling

To determine whether both Notch‐sparing and Notch‐impairing PS1 mutations affect cell viability and cytotoxicity, PS1 variants were transfected into N2AdKO cells. MTS analysis showed that when normalized to PS1_WT_ (cell viability 100%), PS1_∆S169_ had no significant change (97.85 ± 3.498%; *p* > 0.05), while cell viability decreased in cells expressing PS1_M146V_ (82.77 ± 2.15%; *p* < 0.01), PS1_G384A_ (81.58 ± 3.23%; *p* < 0.01), PS1_C410Y_ (70.21 ± 2.85%; *p* < 0.0001), and PS1_Y115H_ (72.41 ± 3.38%; *p* < 0.0001) (Figure 2D).

To assess whether these effects are associated with alterations in Notch signaling, a Hes1 promoter luciferase assay was performed in N2AdKO cells as a functional readout of Notch pathway activity. Cells were co‐transfected with a membrane‐bound Notch fragment (MV), which requires PS1‐mediated cleavage to generate the active NICD, together with a Hes1 reporter construct. PS1_WT_ significantly increased Hes1 activity compared with the PZ (pcDNA4 vector) and MV baseline (*p* < 0.0001). In contrast, PS1_M146V_, PS1_G384A_, PS1_C410Y_, and PS1_Y115H_ showed a significant reduction in Hes1 activity (*p* < 0.001), indicating impaired Notch signaling. PS1_ΔS169_ did not significantly alter Hes1 activity compared with PS1_WT_ (*p* > 0.05), consistent with its classification as a Notch‐sparing mutation. As a positive control, the Notch intracellular domain (ICV plasmid), which bypasses PS1‐mediated processing, was expressed in N2AdKO cells. Co‐expression of ICV restored Hes1 activity to levels comparable to PS1_WT_ (*p* > 0.05) (Figure S4A).

To determine whether impaired Notch signaling contributes to the reduced cell viability observed in PS1 mutant cells, PS1_M146V_ and PS1_C410Y_ were co‐transfected with or without the NICD (ICV plasmid), followed by MTS analysis. PS1_M146V_ and PS1_C410Y_ reduced cell viability compared with PS1_WT_ (*p* < 0.0001). However, co‐expression of ICV with the mutants restored cell viability to levels comparable to PS1_WT_ (*p* > 0.05) (Figure S4B), indicating that reduced viability in these mutants is at least partially mediated by impaired Notch signaling.

On the other hand, to assess the cytotoxic effects of PS1 mutations, LDH release was quantified and expressed as a percentage of cytotoxicity. Compared with PS1_WT_ (8.82 ± 1.05%), all five PS1 mutations led to significantly increased cytotoxicity: 14.27 ± 0.37%, (*p* < 0.001) for PS1_∆S169_, 13.74 ± 0.61% (*p* < 0.001) for PS1_M146V_, 15.88 ± 0.35% (*p* < 0.0001) for PS1_G384A_, 17.71 ± 0.80% (*p* < 0.0001) for PS1_C410Y_, and 15.40 ± 0.17% (*p* < 0.0001) for PS1_Y115H_ (Figure 2E).

### 2.3 PS1 Mutations Do Not Alter BACE1 or BACE1‐Derived APP‐CTF Levels

To determine whether PS1 mutations impact BACE1‐dependent APP cleavage, the same PS1 constructs were transfected into BAW cells, stably expressing APP_WT_ and BACE1 [8], and 2EB2 cells, stably expressing APP_SWE_ and BACE1 [46]. In BAW cells, none of the mutations significantly affected C99 levels (fold changes for PS1_∆S169_: 1.01 ± 0.13, PS1_M146V_: 1.05 ± 0.14, PS1_G384A_: 0.92 ± 0.15, PS1_C410Y_: 0.95 ± 0.17, PS1_Y115H_: 0.81 ± 0.18; *p* > 0.05) (Figure 3A,B). Similarly, C89 levels remained unchanged across all mutants (fold changes range between 0.88 and 1.16; *p* > 0.05) (Figure 3A,C).

**FIGURE 3:**
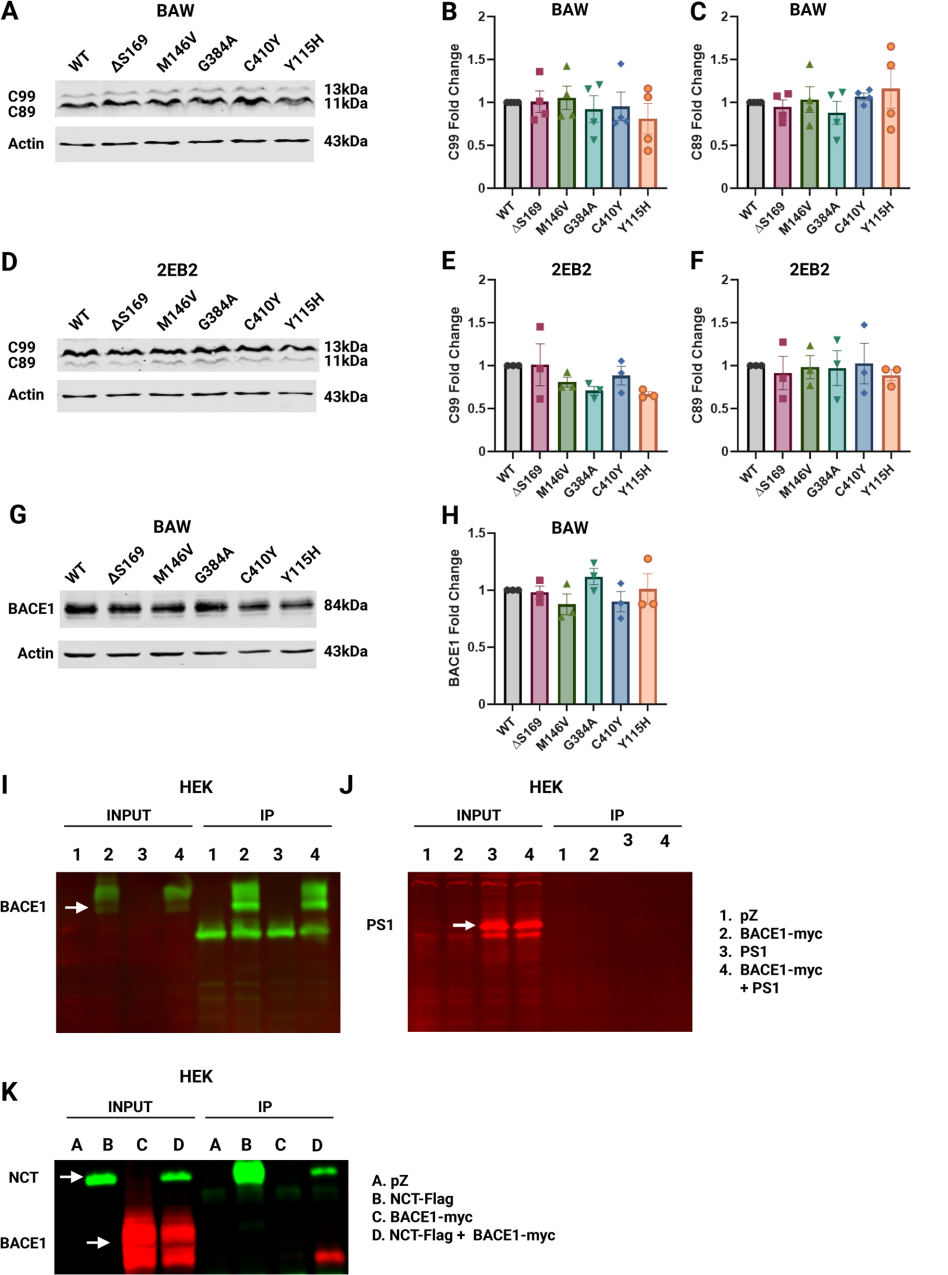
PS1 mutations do not affect BACE1‐mediated APP cleavage or BACE1 protein levels, and PS1 does not directly interact with BACE1. BAW and 2EB2 cells stably overexpressing BACE1 and APP_WT_ or BACE1 and APP_SWE_, respectively, were transfected with PS1_WT_, PS1_ΔS169_, PS1_M146V_, PS1_G384A_, PS1_C410Y_, or PS1_Y115H_. C99 and C89 levels were detected via rabbit anti‐APP antibody C20, while β‐actin was detected by monoclonal mouse anti‐β‐actin antibody. (A) Representative Western blots of C99 and C89 bands in BAW cells; *n* = 4. (B, C) Quantification of C99 and C89 levels in BAW cells. No significant differences were observed; *p* > 0.05. (D) Representative Western blots of C99 and C89 in 2EB2 cells; *n* = 3. (E, F) Quantification of C99 and C89 in 2EB2 cells showed no significant differences between PS1 mutants and PS1_WT_; *p* > 0.05. (G) Representative Western blot for Myc‐tagged BACE1 expression in BAW cells transfected with PS1 variants; *n* = 3. Myc‐tagged BACE1 expression was detected using mouse anti‐Myc 9E10 antibody; β‐actin was detected by monoclonal mouse anti‐β‐actin antibody. (H) Quantification of BACE1 levels showed no significant changes across PS1 mutants compared with PS1_WT_; *p* > 0.05. (I, J) HEK293 cells were co‐transfected with Myc‐tagged BACE1 and PS1. Coimmunoprecipitation (co‐IP) was conducted using anti‐Myc antibodies and blotted with anti‐Myc and anti‐PS1 antibodies. Despite strong expression in the input, PS1 did not coimmunoprecipitate with BACE1. (K) HEK293 cells were co‐transfected with Myc‐tagged BACE1 and FLAG‐tagged Nicastrin (NCT). Cell lysates were subjected to co‐IP using an anti‐Flag antibody, followed by Western blotting with anti‐BACE1 and anti‐Flag antibodies, suggesting that BACE1 directly interacts with NCT, but not with PS1. Approximate molecular weights are indicated: β‐actin (∼43 kDa), C99 (∼13 kDa), C89 (∼11 kDa), myc‐tagged BACE1 (∼84 kDa), FLAG‐tagged NCT (∼110 kDa), PS1 (∼ 46 kDa). Data are shown as mean ± SEM. *p* > 0.05 by one‐way ANOVA.

Under pathological conditions in 2EB2 cells, none of the PS1 mutations significantly altered C99 or C89 levels (fold changes for C99 range from 0.67 to 1.01, and for C89 from 0.89 to 1.03; all *p* > 0.05) (Figure 3D–F). Furthermore, the expression levels of Myc‐tagged BACE1 in BAW cells were unaffected by any of the mutations, with fold changes ranging between 0.87 and 1.12 (*p* > 0.05) (Figure 3G,H). Co‐IP assays revealed that PS1 does not directly interact with BACE1 (Figure 3I,J), whereas Nicastrin (NCT), a component of the γ‐secretase complex, successfully co‐precipitated BACE1 (Figure 3K). Together, these results suggest that PS1 does not directly interact with BACE1, and that mutations in PS1 do not alter BACE1 levels or BACE1‐mediated processing of APP.

APP‐FL levels were also assessed across BAW and 2EB2 cell lines following transfection of PS1 variants. No significant differences in APP‐FL expression were observed between any of the PS1 mutants and PS1_WT_ in either cell line (*p* > 0.05) (Figure S1C–F).

To further determine whether other proteins involved in APP processing were affected by the PS1 mutations, levels of α‐secretase‐related proteins (ADAM10 and ADAM17) and PS2 were quantified in BAW and 2EB2 cells after transfection of PS1 variants. No significant differences were detected for any of these proteins across PS1 mutations compared with PS1_WT_ (*p* > 0.05) (Figure S2G–R). In addition, representative images for PS1 full‐length (PS1‐FL) and processed fragments (PS1‐NTF and CTF) across 20E2, BAW, and 2EB2 cells are shown in Figure S5A–C.

### 2.4 PS1 Mutations Differentially Affect Endoproteolysis, With Reduced Endoproteolysis Associated With Increased C83 Levels

To examine whether PS1 pathological mutations alter their endoproteolytic activity, the N‐terminal fragment (NTF) to full‐length (FL) PS1 ratio was measured in N2AdKO cells co‐transfected with C99 and PS1 variants. Full Western blot images with annotated molecular weights are provided in Figure S6A,B. Total PS1‐FL levels were not significantly altered; fold changes range from 0.88 to 0.99; *p* > 0.05 (Figure 4A,B). Similarly, PS1‐NTF levels were unchanged (*p* > 0.05) for most mutations except for PS1_C410Y_, which showed a significant decrease (fold change of 0.21 ± 0.06; *p* < 0.0001) compared with PS1_WT_ (Figure 4A,C).

**FIGURE 4:**
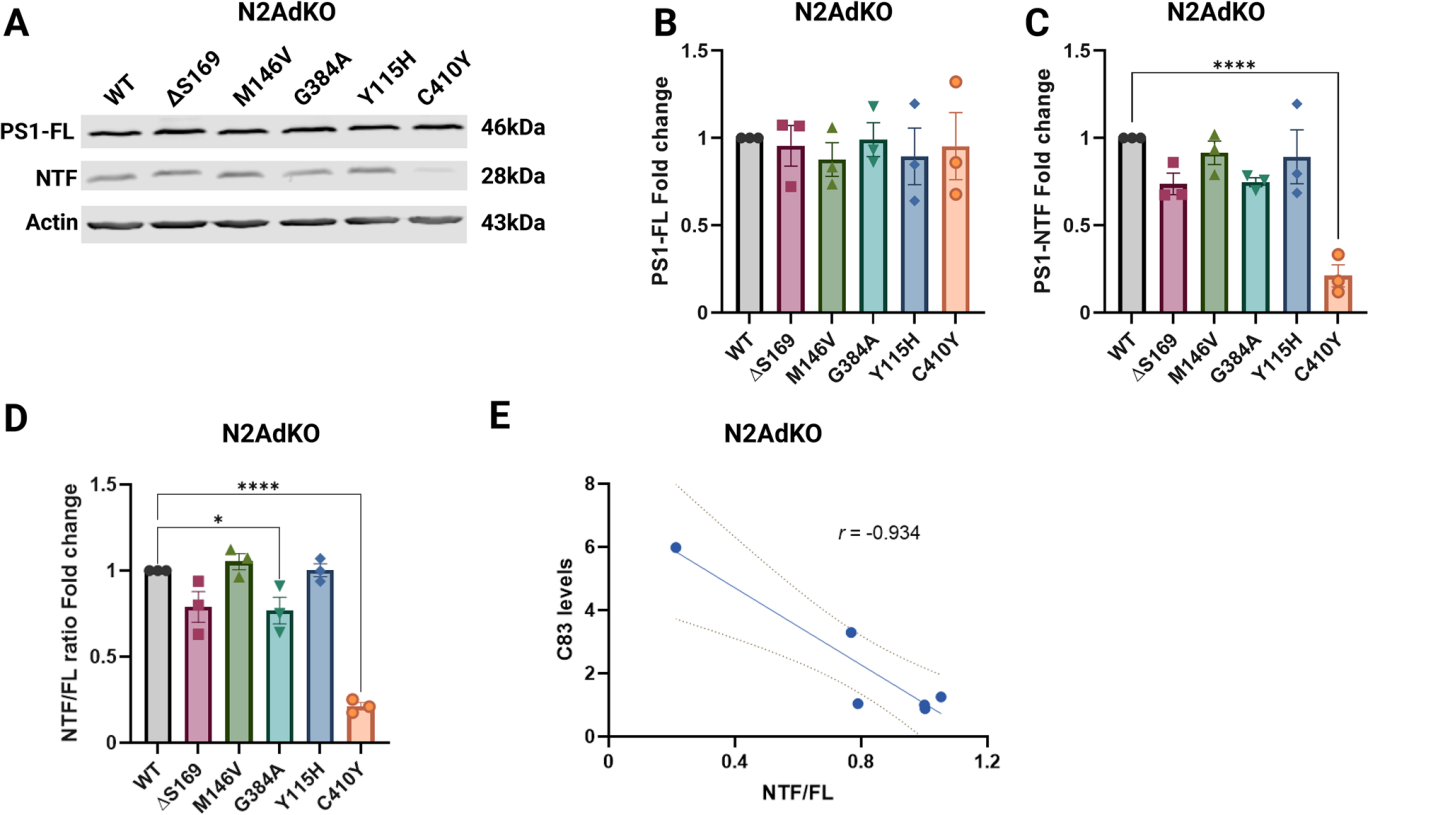
PS1 mutations affect the rate of PS1 endoproteolysis, and this rate correlates with C83 accumulation. N2AdKO cells were co‐transfected with C99 and PS1 variants, including PS1_WT_, PS1_ΔS169_, PS1_M146V_, PS1_G384A_, PS1_C410Y_, and PS1_Y115H_. (A) Representative Western blots for full‐length (FL) PS1 and PS1 N‐terminal fragment (NTF) measured using rabbit PS1N antibody and mouse monoclonal β‐actin antibody; *n* = 3. (B, C) Quantification of FL and NTF levels normalized to PS1_WT_. Only PS1_C410Y_ significantly reduced NTF levels; ****p < 0.0001. (D) Quantification of the NTF/FL ratio as a measure of endoproteolytic efficiency. PS1_G384A_ and PS1_C410Y_ showed significantly reduced endoproteolysis; *p < 0.05, ****p < 0.0001. (E) Correlation analysis between NTF/FL ratio and C83 levels revealed a significant negative correlation (*r* = −0.934, *R* ^2^ = 0.87, **p < 0.01). Dotted lines showed 95% confidence bands. Approximate molecular weights are indicated: β‐actin (∼43 kDa), PS1‐FL (∼46 kDa), and PS1‐NTF (∼28 kDa). Data are shown as mean ± SEM, *n* = 3. Statistical analyses were completed by one‐way ANOVA followed by Dunnett's post hoc test or Pearson correlation.

Endoproteolytic efficiency, as assessed by the NTF/FL ratio, was significantly reduced compared with PS1_WT_ for PS1_G384A_ (fold change of 0.77 ± 0.078; *p* < 0.05) and PS1_C410Y_ (0.21 ± 0.02; *p* < 0.0001), while PS1_∆S169_, PS1_M146V_, and PS1_Y115H_ showed no significant change (fold changes range from 0.79 to 1.05; *p* > 0.05) (Figure 4A,D). Pearson correlation analysis revealed no significant correlation (*p* > 0.05) between endoproteolytic rate and Aβ40 levels, Aβ42 levels, the Aβ42/Aβ40 ratio, or C99 levels (*r* = 0.632, *R* ^2^ = 0.399, *p* > 0.05; *r* = 0.603, *R* ^2^ = 0.363, *p* > 0.05; *r* = 0.018, *R* ^2^ = 0.0003, *p* > 0.05; *r* = 0.0378, *R* ^2^ = 0.0014, *p* > 0.05, respectively) (Figure S7A–D). Importantly, a strong negative correlation (*r* = −0.934, *R^2^* = 0.87, *p* < 0.01) was found between the NTF/FL ratio and C83 levels (Figure 4E), indicating that reduced PS1 endoproteolysis correlates with increased C83 accumulation in N2AdKO cells.

## 3 Discussion

In this study, we investigated the effects of five FAD‐associated PS1 mutations on APP processing, Aβ production, cytotoxicity, and PS1 endoproteolysis. Using four cell lines reflecting both physiological and pathological conditions, we found that these mutations can differentially affect APP cleavage and cytotoxicity, elevate the Aβ42/Aβ40 ratio, and, in some cases, impair PS1 endoproteolysis.

All five PS1 mutations significantly increased C83 levels in 20E2 cells, which overexpress the pathogenic APP_SWE_ variant [45]. These findings extend our previous work in HAW cells expressing wild‐type APP (APP_WT_), where both Notch‐sparing and Notch‐impairing mutations similarly elevated C83 [13]. Importantly, in N2AdKO cells transfected with C99, only PS1_G384A_ and PS1_C410Y_ showed a significant elevation in C83 levels. Since levels of ADAM10 and ADAM17, both key α‐secretases, were not altered by PS1 mutations in any of the cell lines, the observed increase in C83 is unlikely to result from enhanced production. Instead, it is more likely to reflect reduced γ‐secretase‐mediated processing of C83, an effect that appears to be substrate‐specific, as it is not observed for C99.

The differences in C83 accumulation between HAW, 20E2, and N2AdKO cells may arise from several factors. These include species‐ or cell‐specific effects, particularly the differences between human embryonic kidney cells and mouse neuroblastoma cells, as well as the varying contributions of endogenous versus exogenous PS1 and PS2 to γ‐secretase cleavage. Additionally, the type of APP species expressed (APP_WT_, APP_SWE_, or C99) may also play a role. These variables likely influence how each mutation affects γ‐secretase processing.

C83 accumulation can be interpreted as a consequence of impaired γ‐secretase cleavage of the α‐secretase product. Although often considered less toxic than C99 or Aβ, C83 has been implicated in ER (endoplasmic reticulum) stress‐induced cell death [47]. Moreover, elevated C83 levels have been observed in AD patient brains [48]. Similarly, APP‐CTF accumulation, including that of C83, has been shown to impair mitochondrial morphology and function [48]. Our results suggest that C83 accumulation is a shared feature of several FAD‐linked PS1 mutations in vitro. Importantly, our findings suggest that C83 accumulation might not only be a byproduct of potentially impaired γ‐secretase activity but also may contribute directly to cellular dysfunction. The selective correlation between impaired PS1 endoproteolysis and increased C83, but not C99, further indicates that α‐secretase‐derived substrates may be differentially affected by PS1 dysfunction. Together, these results highlight C83 as a potential contributor to AD‐related pathology.

Aβ40 is the most common Aβ form, while Aβ42 can be more prone to aggregation and thus is the primary form found in neuritic plaques, contributing to neurodegeneration [49]. Thus, the ratio of Aβ42/Aβ40 is a crucial clinical biomarker in AD. In our study, all five PS1 mutations significantly increased the Aβ42/Aβ40 ratio in N2AdKO cells. However, the specific changes in Aβ40 and Aβ42 levels varied by mutation. While PS1_∆S169_, PS1_M146V_, and PS1_Y115H_ increased Aβ42 and decreased Aβ40, PS1_G384A_ and PS1_C410Y_ significantly reduced both Aβ forms, with Aβ40 showing a steeper decline.

These findings build upon our earlier research that focused on three specific mutations (PS1_∆S169_, PS1_Y115H_ and PS1_C410Y_) in PS1/PS2‐knockout mouse fibroblast cells [13]. They are also consistent with previous reports on mutation‐specific Aβ levels [39, 44]. Notably, mutations like PS1_ΔS169_ may skew the γ‐secretase cleavage process toward producing longer and more pathogenic forms, such as Aβ42, without broadly disrupting overall cleavage efficiency. In contrast, mutations like PS1_C410Y_ may significantly impair γ‐secretase activity, resulting in a decrease in total Aβ production.

This interpretation is supported by previous studies showing that PS1_C410Y_ can severely impair γ‐secretase activity while still resulting in pathological changes [41, 50]. Additionally, PS1 mutations may disrupt the initial ε‐cleavage step or change the preferred γ‐cleavage site, thereby altering Aβ levels [38]. The consistent elevation of the Aβ42/Aβ40 ratio across all examined mutations, despite the changes in specific Aβ levels, reinforces the idea that this ratio serves as a common hallmark of pathogenic PS1 mutations. It also highlights the value of the Aβ42/Aβ40 ratio in both diagnostic and mechanistic studies.

Whether PS1 mutations directly affect BACE1 function remains a topic of debate. Some studies report altered BACE1 trafficking, expression, or activity in the context of PS1 mutations [51, 52]. Conversely, other research finds no impact of PS1 mutations on BACE1‐mediated cleavage of APP [53], and analyses of cerebrospinal fluid and brain samples from FAD patients, including those with PS1 mutations, show no significant changes in BACE1 levels or activity compared with controls [54]. To address this, we measured C99 and C89, direct products of BACE1 cleavage, in different cell lines. Across both physiological APP_WT_ (BAW) and pathological APP_SWE_ (2EB2) cell lines, no significant changes were observed in C99, C89, or BACE1 levels upon expression of PS1 mutants. Similarly, C99 levels in the N2AdKO cells transfected with the PS1 mutations were unchanged across all five mutations. Furthermore, co‐IP experiments revealed no detectable direct interaction between PS1 and BACE1, although NCT (a γ‐secretase subunit) co‐precipitated with BACE1, consistent with previous reports [55]. Collectively, these results suggest that the five PS1 mutations studied do not influence APP processing via BACE1. This reinforces γ‐secretase dysfunction as a key pathogenic mechanism in FAD‐PS1 mutations.

A key finding from this study is that specific PS1 mutations impair PS1 endoproteolysis, which generates stable PS1‐NTFs and ‐CTFs [11, 15, 16, 17, 56]. The role and importance of this endoproteolytic step remain debated. Our results showed that PS1_G384A_ and PS1_C410Y_ significantly reduced the NTF/FL ratio, indicating impaired endoproteolytic maturation. This was not observed with PS1_∆S169_, PS1_M146V_, or PS1_Y115H_ mutations. The location of the mutations may influence whether they inhibit endoproteolysis. For instance, mutations located near the endoproteolytic cleavage site, around Met298 [56], are thought to impair PS1 processing. Notably, even when PS1 remains uncleaved, the catalytic pore of γ‐secretase can still form, allowing partial enzymatic activity to persist [57]. This partial activity may explain the altered Aβ production observed in mutations near this endoproteolytic site [57, 58]. However, in this study, PS1_G384A_ and PS1_C410Y_ were the only two mutations that both impaired endoproteolysis and reduced levels of Aβ40 and Aβ42, despite being located downstream of the cleavage site. This supports the idea that while γ‐secretase can remain active in the absence of PS1 endoproteolysis, its efficiency is compromised, leading to lower Aβ production. Furthermore, endoproteolysis is influenced not only by the mutation site, but also by interactions with other γ‐secretase subunits [59, 60, 61, 62]. Disrupted interactions with NCT, anterior pharynx‐defective 1 (APH‐1) or presenilin enhancer 2 (PEN2) can destabilize PS1 or alter its catalytic activity [57, 59, 60, 61, 62]. Potentially, PS1_G384A_ and PS1_C410Y_ could be affecting these interactions, leading to inhibited endoproteolysis. Taken together, these findings suggest that mutation‐specific effects on PS1 endoproteolysis might be mediated by altered interactions within the γ‐secretase complex or changes in PS1 structural stability, rather than solely by proximity to the cleavage site.

Although no correlation was found between the endoproteolysis rate (NTF/FL) and the levels of Aβ40, Aβ42, or their ratio, we observed a strong negative correlation between the NTF/FL and C83 levels. This suggests that impaired processing of PS1 might lead to reduced γ‐secretase activity and substrate accumulation. One possibility behind this correlation is that reduced endoproteolysis may impact the assembly of the γ‐secretase complex, thereby altering its ability to engage and cleave CTFα and C83. The structural integrity of each subunit depends on its association with the others, and a PS1 mutation (for instance, PS1_ΔE9_, which inhibits endoproteolysis [18, 63]) can alter the conformation of the γ‐secretase complex [64]. Thus, mutations such as PS1_G384A_ and PS1_C410Y_, which also reduce endoproteolysis, may also induce conformational changes that disrupt subunit interactions. These shifts in conformation can selectively impair the processing of certain substrates without completely abolishing catalytic activity. The correlation between PS1 endoproteolysis and C83, but not C99, suggests that α‐secretase‐derived substrates may be differentially affected by alterations in γ‐secretase function. This may reflect differences in substrate engagement or processing efficiency within the complex. Together, these findings indicate that PS1‐related γ‐secretase dysfunction does not uniformly affect all substrates and support the existence of substrate‐specific regulatory mechanisms.

In this study, we conducted MTS and LDH assays to assess cell viability and cytotoxicity in N2AdKO cells expressing the PS1 mutations. Our results showed that most PS1 mutations led to compromised cell viability and all increased cytotoxicity. Specifically, all mutations, except for PS1_ΔS169_, resulted in a significant reduction in MTS‐based cell viability. Additionally, the LDH assays indicated increased LDH release linked to membrane damage across all PS1 variants.

Notch signaling is crucial for regulating cell fate, proliferation, and survival, while dysregulation of this pathway has been linked to decreased viability and increased apoptosis in various models [24, 25, 26, 65]. Since PS1 is required for the cleavage of Notch receptors [9, 11, 66], impaired Notch processing may explain the reduced viability observed in cells with PS1 mutations that affect Notch function. The absence of significant viability loss in PS1_ΔS169_, which spares Notch, supports this hypothesis. Importantly, our Hes1 luciferase data further support this conclusion by providing a direct functional readout of Notch signaling. Mutations that reduced cell viability (PS1_M146V_, PS1_G384A_, PS1_C410Y_, and PS1_Y115H_) also showed reduced Hes1 activity, whereas PS1Δ_S169_ did not, consistent with preserved Notch signaling and previous reports [13]. Furthermore, our experiments using the Notch intracellular domain (NICD, plasmid ICV) showed that restoring Notch signaling rescues the reduced cell viability observed in PS1_M146V_ and PS1_C410Y_ mutants. This provides evidence that impaired Notch signaling contributes to the decreased viability associated with these mutations. However, the Notch pathway does not seem to be responsible for the cytotoxicity indicated by LDH release, as all mutant cells, including those with PS1_ΔS169_, exhibited comparable levels of membrane damage‐linked cytotoxicity.

An additional explanation for the discrepancy between the MTS and LDH results might be related to the different processes that each test measures. The MTS assay assesses metabolic activity, looking into mitochondrial function and overall cell viability [67]. In contrast, LDH release is linked to loss of membrane integrity and cytotoxicity [68]. These differences suggest that specific PS1 mutations may lead to membrane damage without significantly affecting mitochondrial function. Overall, these findings highlight that PS1 mutations can have specific effects on cell health, and that this effect might be mediated through pathways involving Notch signaling, mitochondrial function, or membrane integrity.

While this study offers an in‐depth analysis of FAD‐linked PS1 mutations, several limitations should be considered. Only five PS1 mutations were examined; therefore, the effects may not be representative of other PS1 mutations. Additionally, cell models may not fully capture the impact of PS1 mutations in the human brain, including neuronal network interactions, glial contributions, and the influence of the blood‐brain barrier on these effects. Future studies using iPSC‐derived neurons from patients carrying PS1 mutations will allow validation of these findings in a more physiologically relevant human model. In addition, transgenic mouse models, such as APP/PS1, could be used to examine the in vivo effects of the mutations on C83 levels, Aβ plaques, and associated neuroinflammatory responses. Importantly, the long‐term consequences of C83 accumulation were not addressed in this study. Chronic elevation of C83 may disrupt ER and mitochondrial function, and long‐term culture systems or in vivo models will be necessary to determine whether sustained accumulation contributes to neurodegeneration, ER stress, or mitochondrial dysfunction. Future studies could further define the mechanisms of C83‐mediated toxicity by using C83‐overexpression models and identifying C83‐interacting proteins. These approaches may help clarify how C83 accumulation contributes to downstream cellular stress pathways such as ER stress and mitochondrial dysfunction. In addition, structural studies may help define how specific PS1 mutations alter substrate cleavage or γ‐secretase complex assembly. Lastly, the NTF/FL ratio used in this study represents a steady‐state measure of PS1 endoproteolysis and may not fully capture the dynamic processes underlying PS1 maturation. Changes in this ratio may reflect not only altered cleavage efficiency but also differences in fragment stability or degradation. For example, reduced NTF levels could result from impaired endoproteolysis or increased degradation of the fragment. Future studies using approaches such as pulse‐chase labeling or inhibition of proteasomal and lysosomal pathways will be required to distinguish between these possibilities and to more fully define the mechanisms underlying altered PS1 processing.

## 4 Conclusion

In summary, this study demonstrates that FAD‐causing PS1 mutations can affect APP processing, Aβ production, and PS1 endoproteolysis differently. Despite these mutation‐specific differences, all examined PS1 mutations lead to an increase in the Aβ42/Aβ40 ratio, a key pathogenic hallmark of AD. Our findings also reveal a potential novel correlation between impaired PS1 endoproteolysis and APP‐C83 accumulation, suggesting a mechanistic link between PS1 endoproteolysis and substrate cleavage. Furthermore, most PS1 mutations reduced cell viability, which is at least partially mediated by impaired Notch signaling, while increased cytotoxicity appears to involve additional Notch‐independent mechanisms. Overall, these results further our understanding of how various PS1 mutations contribute to AD pathogenesis and may inform future therapeutic strategies.

## 5 Materials and Methods

### 5.1 Plasmid Constructs

Plasmids encoding presenilin 1 wild‐type (PS1_WT_), PS1_∆S169_, PS1_M146V_, PS1_G384A_, PS1_C410Y_, and PS1_Y115H_ were cloned into pcDNA4‐Myc‐His (A) vector [11, 13]. Additionally, APP C99 was cloned into the pcDNA4‐Myc‐His (A) vector to generate the plasmid pAPP‐C99 construct [45]. Plasmid MV, a membrane‐bound Notch fragment, requires PS1 cleavage to initiate Notch. Plasmid ICV expresses the truncated form of NΔE from the cleavage site 1744 corresponding to the Notch‐1 intracellular domain (NICD) [11, 13]. Lastly, NCT‐Flag and BACE1‐Myc plasmids were used in the coimmunoprecipitation experiments [69, 70, 71].

### 5.2 Cell Culture and Transfection

All cell lines were maintained at 37°C in an incubator with 5% CO_2_. The N2AdKO cell line (Sigma #SCC297), a PS1/PS2 double knockout mouse neuroblastoma (N2A) line, was cultured in media according to the manufacturer's protocol. Human embryonic kidney (HEK293) cells and stable HEK293 cell lines, including 20E2, BAW, and 2EB2 [7, 8], were maintained in a medium consisting of 90% Dulbecco's modified Eagle medium (DMEM) (Fisher Scientific #SH30243.01) with 10% FBS (Fisher Scientific #12483‐028), and supplemented with Zeocin (Thermo Scientific #R25001) and/or G418 (Sigma #A1720‐5G). Plasmid transfections were carried out using Lipofectamine 3000 (Thermo Scientific #L3000015) according to the manufacturer's instructions. All cell lines tested negative for mycoplasma contamination and were analyzed with Short Tandem Repeat to confirm their identity.

### 5.3 Immunoblotting and Immunoprecipitation

Cells were lysed in RIPA buffer (Thermo Scientific #89901) supplemented with a protease inhibitor (Thermo Scientific #78429), followed by sonication and centrifugation at 13,200 rpm for 15 min at 4°C. Protein samples were mixed with 4xSDS sample buffer and boiled at 100°C for 5 min. The samples were then separated using 10% Tris‐glycine sodium dodecyl sulphate‐polyacrylamide (SDS‐PAGE) or 16% Tris‐tricine SDS‐PAGE and transferred to nitrocellulose membranes (GVS North America #1215471). Membranes were blocked with 5% nonfat dried milk (Bio‐Rad #1706404) in Tris‐buffered saline (TBS). They were then incubated overnight at 4°C with blocking buffer containing 0.1% Tween 20 (TBST) and primary antibodies. The primary antibodies used included C20 (1:1000) [46] for detecting APP and its C‐terminal fragments (CTF), PS1N (1:5000) [13] for full‐length PS1 and its N‐terminal fragment (NTF), and 9E10 (1:100) [46] for Myc‐tag detection. The following day, membranes were incubated for 1 h with β‐actin antibody, using either a mouse antibody (1:10,000) (Sigma #A1978) or a rabbit antibody (1:500) (Abcam #ab115777). Membranes were then incubated with appropriate secondary antibodies, including IRDye 680RD goat anti‐mouse (1:20,000) (LI‐COR Biosciences #926‐68070) and IRDye 800CW goat anti‐rabbit (1:15,000) (LI‐COR Biosciences #926‐32211) for 1 h at room temperature. Visualization was performed using the Odyssey system (LI‐COR Biosciences). Quantifications were conducted using the LI‐COR Empiria Studio system.

For the detection of ADAM10, ADAM17, PS2, and PS1‐CTF, membranes were developed using enhanced chemiluminescence (ECL). Primary antibodies included human ADAM17 (Santa Cruz #SC‐390859, 1:100) for HEK‐derived stable cells, mouse ADAM17 (Abcam #ab39126, 1:1000) for N2AdKO cells, ADAM10 (Abcam #ab124695, 1:1000), PS2 (Abcam #ab51249, 1:2000), and PS1 (Abcam #ab76083, 1:2000). Corresponding HRP‐conjugated secondary antibodies (Goat anti‐mouse, ZSGB‐Bio #ZB‐2305; Goat anti‐rabbit, ZSGB‐Bio #ZB‐2301; both 1:10,000) were used for signal detection.

In the coimmunoprecipitation (co‐IP) experiment, the BACE1‐Myc plasmid was co‐transfected with PS1 or NCT (Nicastrin)‐Flag plasmids into HEK293 cells by lipofectamine 2000 (Invitrogen #11668019). Forty‐eight hours posttransfection, cells were harvested in lysis buffer (Beyotime #P0013) supplemented with protease inhibitor cocktail (Beyotime #P1005). For precipitations with anti‐Myc (for BACE1, Sigma #M5546) or anti‐Flag (for NCT), the antibodies were first incubated with protein A/G beads (Santa Cruz #sc‐2003) for at least 3 h at 4°C with rotation, then added to cell lysates. Lysates were then incubated with antibody‐conjugated beads at 4°C with rotation overnight. The following day, three washes with lysis buffer were done to remove unbound proteins. Precipitated proteins were then eluted from the beads by 1× SDS sample buffer (Beyotime #P0015) at room temperature for 30 min (for BACE1‐PS1 co‐IP) or by boiling in 1× SDS sample buffer (for BACE1‐NCT co‐IP) for 5–10 min. Proteins were resolved by SDS‐PAGE and blotted with anti‐Myc antibody to detect BACE1 (Sigma #M5546, 1:200), and anti‐PS1 antibody for PS1 (Abcam #EP2000Y, 1:2000) for the BACE1‐PS1 co‐IP, while using anti‐BACE1 antibody to detect BACE1 (Abcam #ab108394, 1:2000), and anti‐flag antibody for NCT (Sigma # F3165, 1:5,000).

### 5.4 Aβ40/42 Enzyme‐Linked Immunosorbent Assay (ELISA)

Conditioned medium was collected 48 h after transfection. The medium was then centrifuged at 13,200 rpm for 2 min at 4°C and treated with protease inhibitors containing AEBSF (Thermo Scientific #78429). The concentrations of Aβ40 and Aβ42 were measured using β‐amyloid 1–40 (Thermo Scientific #KHB3481) and β‐amyloid 1–42 (Thermo Scientific #KHB3441) ELISA kits following the manufacturer's protocols.

### 5.5 MTS and LDH Assays

N2AdKO cells were seeded into 96‐well plates and then transfected using Lipofectamine 3000 (Thermo Scientific #L3000015) with PS1 variants. To detect cell viability, MTS assays (Abcam #ab197010) were performed according to the manufacturer's recommendations. Similarly, LDH assays (Abcam #ab65393) were completed using conditioned media from the N2AdKO‐transfected cells to detect cytotoxicity according to the manufacturer's protocol.

### 5.6 Luciferase Assay

N2AdKO cells were seeded into 24‐well plates and transfected the following day using Lipofectamine 3000 (Thermo Scientific #L3000015) according to the manufacturer's instructions. Forty‐eight hours posttransfection, cells were lysed in 100 µL of 1× passive lysis buffer (Promega) per well. Firefly and Renilla luciferase activities were measured sequentially using the dual‐luciferase reporter assay system (Promega #E1910) following the manufacturer's protocol. Firefly luciferase activity was normalized to Renilla luciferase activity and expressed as relative luciferase units (RLU) to reflect promoter activity.

### 5.7 Statistical Analysis

Statistical analyses were performed using GraphPad Prism version 10.5. Data are presented as mean ± standard error of the mean (SEM). Each experiment included at least three replicates, as noted in the figure legends. One‐way analysis of variance (ANOVA) followed by Dunnett's multiple comparisons test was used for group comparisons. Pearson correlation coefficients were calculated for correlation analyses. A *p*‐value < 0.05 was considered statistically significant.

## Author Contributions

I.B.L. and W.S. conceived and designed the experiments. I.B.L., K.S., L.L., Z.W., Q.Z., and F.C. performed the experiments. I.B.L., K.S., L.L., Z.L., Z.W., Q.Z., W.P., F.C., and W.S. analyzed the data and/or contributed materials and analytical tools. I.B.L. drafted the manuscript and prepared the figures. W.S. and F.C. revised the manuscript. All authors reviewed and approved the final manuscript.

## Funding

This work was supported by the Canadian Institutes of Health Research (CIHR) (PJT‐166127); Canada Foundation for Innovation/John R. Evans Leaders Fund—Partnerships (38460); National Natural Science Foundation of China (82293642, 82230043); Department of Science and Technology of Zhejiang Province (2023ZY1018, 2024SSYS0054); Oujiang Lab (OJQDJQ2022002). IBL was supported by the Four‐Year Fellowship from the University of British Columbia and the Jock and Irene Graham Brain Endowment Award.

## Conflicts of Interest

Weihong Song is an Editorial Board member of *MedComm*. Dr. Song was not involved in the journal's review or decisions related to this manuscript. The remaining authors declare no conflicts of interest.

## Ethics Statement

The authors have nothing to report.

## Supporting information




**Supporting File 1**: mco270978‐sup‐0001‐SuppMat.docx

## Data Availability

All data are available in the manuscript or Supporting Information.

## References

[1] Y. Zhang , H. Chen , R. Li , K. Sterling , and W. Song , “Amyloid β‐Based Therapy for Alzheimer's Disease: Challenges, Successes and Future,” Signal Transduction Target Therapy 8, no. 1 (2023): 248. 10.1038/s41392-023-01484-7 PMC10310781 37386015

[2] Y. Zhang and W. Song , “Islet Amyloid Polypeptide: Another Key Molecule in Alzheimer's Pathogenesis?,” Progress in Neurobiology 153 (2017): 100–120. 10.1016/j.pneurobio.2017.03.001 28274676

[3] F. S. Esch , P. S. Keim , E. C. Beattie , et al., “Cleavage of Amyloid Beta Peptide During Constitutive Processing of Its Precursor,” Science 248, no. 4959 (1990): 1122–1124. 10.1126/science.2111583 2111583

[4] J. D. Buxbaum , K. N. Liu , Y. Luo , et al., “Evidence That Tumor Necrosis Factor Alpha Converting Enzyme Is Involved in Regulated Alpha‐secretase Cleavage of the Alzheimer Amyloid Protein Precursor,” Journal of Biological Chemistry 273, no. 43 (1998): 27765–27767. 10.1074/jbc.273.43.27765 9774383

[5] Q. Zhang , M. Xing , Z. Bao , et al., “Contactin‐associated Protein‐Like 2 (CNTNAP2) Mutations Impair the Essential Alpha‐Secretase Cleavages, Leading to Autism‐Like Phenotypes,” Signal Transduction Target Therapy 9, no. 1 (2024): 51. 10.1038/s41392-024-01768-6 PMC10904759 38424048

[6] Y. Li , W. Zhou , Y. Tong , G. He , and W. Song , “Control of APP Processing and Abeta Generation Level by BACE1 Enzymatic Activity and Transcription,” Faseb Journal 20, no. 2 (2006): 285–292. 10.1096/fj.05-4986com 16449801

[7] S. Zhang , Z. Wang , F. Cai , et al., “BACE1 Cleavage Site Selection Critical for Amyloidogenesis and Alzheimer's Pathogenesis,” Journal of Neuroscience 37, no. 29 (2017): 6915–6925. 10.1523/JNEUROSCI.0340-17.2017 PMC6705717 28626014

[8] Y. Deng , Z. Wang , R. Wang , et al., “Amyloid‐β Protein (Aβ) Glu11 Is the Major β‐secretase Site of β‐Site Amyloid‐β Precursor Protein‐Cleaving Enzyme 1(BACE1), and Shifting the Cleavage Site to Aβ Asp1 Contributes to Alzheimer Pathogenesis,” European Journal of Neuroscience 37, no. 12 (2013): 1962–1969. 10.1111/ejn.12235 23773065

[9] Z. Zhang , P. Nadeau , W. Song , et al., “Presenilins Are Required for Gamma‐Secretase Cleavage of Beta‐APP and Transmembrane Cleavage of Notch‐1,” Nature Cell Biology 2, no. 7 (2000): 463–465. 10.1038/35017108 10878814

[10] B. De Strooper , “Lessons From a Failed γ‐Secretase Alzheimer Trial,” Cell 159, no. 4 (2014): 721–726. 10.1016/j.cell.2014.10.016 25417150

[11] W. Song , P. Nadeau , M. Yuan , X. Yang , J. Shen , and B. A. Yankner , “Proteolytic Release and Nuclear Translocation of Notch‐1 Are Induced by Presenilin‐1 and Impaired by Pathogenic Presenilin‐1 Mutations,” PNAS 96, no. 12 (1999): 6959–6963. 10.1073/pnas.96.12.6959 PMC22024 10359821

[12] R. S. Doody , R. Raman , M. Farlow , et al., “A Phase 3 Trial of semagacestat for Treatment of Alzheimer's Disease,” New England Journal of Medicine 369, no. 4 (2013): 341–350. 10.1056/NEJMoa1210951 23883379

[13] S. Zhang , F. Cai , Y. Wu , et al., “A Presenilin‐1 Mutation Causes Alzheimer Disease Without Affecting Notch Signaling,” Molecular Psychiatry 25, no. 3 (2020): 603–613. 10.1038/s41380-018-0101-x 29915376

[14] S. Zhang , M. Zhang , F. Cai , and W. Song , “Biological Function of Presenilin and Its Role in AD Pathogenesis,” Translational Neurodegeneration 2, no. 1 (2013): 15. 10.1186/2047-9158-2-15 PMC3718700 23866842

[15] D. Levitan , J. Lee , L. Song , et al., “PS1 N‐ and C‐terminal Fragments Form a Complex That Functions in APP Processing and Notch Signaling,” Proceedings of National Academy of Sciences 98, no. 21 (2001): 12186–12190. 10.1073/pnas.211321898 PMC59789 11593035

[16] M. S. Wolfe , W. Xia , B. L. Ostaszewski , T. S. Diehl , W. T. Kimberly , and D. J. Selkoe , “Two Transmembrane Aspartates in Presenilin‐1 Required for Presenilin Endoproteolysis and Gamma‐Secretase Activity,” Nature 398, no. 6727 (1999): 513–517. 10.1038/19077 10206644

[17] G. Thinakaran , D. R. Borchelt , M. K. Lee , et al., “Endoproteolysis of Presenilin 1 and Accumulation of Processed Derivatives in Vivo,” Neuron 17, no. 1 (1996): 181–190. 10.1016/s0896-6273(00)80291-3 8755489

[18] H. Steiner , H. Romig , M. G. Grim , et al., “The Biological and Pathological Function of the Presenilin‐1 Deltaexon 9 Mutation Is Independent of Its Defect to Undergo Proteolytic Processing,” Journal of Biological Chemistry 274, no. 12 (1999): 7615–7618. 10.1074/jbc.274.12.7615 10075646

[19] A. Capell , H. Steiner , H. Romig , et al., “Presenilin‐1 Differentially Facilitates Endoproteolysis of the Beta‐Amyloid Precursor Protein and Notch,” Nature Cell Biology 2, no. 4 (2000): 205–211. 10.1038/35008626 10783238

[20] M. Grilli , E. Diodato , G. Lozza , et al., “Presenilin‐1 Regulates the Neuronal Threshold to Excitotoxicity both Physiologically and Pathologically,” Proceedings of National Academy of Sciences 97, no. 23 (2000): 12822–12827. 10.1073/pnas.97.23.12822 PMC18848 11070093

[21] F. Terro , C. Czech , F. Esclaire , et al., “Neurons Overexpressing Mutant Presenilin‐1 Are More Sensitive to Apoptosis Induced by Endoplasmic Reticulum‐Golgi Stress,” Journal of Neuroscience Research 69, no. 4 (2002): 530–539. 10.1002/jnr.10312 12210846

[22] J. Han , H. Park , C. Maharana , et al., “Alzheimer's Disease‐Causing Presenilin‐1 Mutations Have Deleterious Effects on Mitochondrial Function,” Theranostics 11, no. 18 (2021): 8855–8873. 10.7150/thno.59776 PMC8419044 34522215

[23] L. Rojas‐Charry , S. Calero‐Martinez , C. Morganti , et al., “Susceptibility to Cellular Stress in PS1 Mutant N2a Cells Is Associated With Mitochondrial Defects and Altered Calcium Homeostasis,” Scientific Reports 10, no. 1 (2020): 6455. 10.1038/s41598-020-63254-7 PMC7160112 32296078

[24] S. Artavanis‐Tsakonas , M. D. Rand , and R. J. Lake , “Notch Signaling: Cell Fate Control and Signal Integration in Development,” Science 284, no. 5415 (1999): 770–776. 10.1126/science.284.5415.770 10221902

[25] S. Lowell , A. Benchoua , B. Heavey , and A. G. Smith , “Notch Promotes Neural Lineage Entry by Pluripotent Embryonic Stem Cells,” PLoS Biology 4, no. 5 (2006): e121. 10.1371/journal.pbio.0040121 PMC1431581 16594731

[26] J. H. van Es , M. E. van Gijn , O. Riccio , et al., “Notch/Gamma‐Secretase Inhibition Turns Proliferative Cells in Intestinal Crypts and Adenomas Into Goblet Cells,” Nature 435, no. 7044 (2005): 959–963. 10.1038/nature03659 15959515

[27] J. Guo , J. Wei , S. Liao , L. Wang , H. Jiang , and B. Tang , “A Novel Presenilin 1 Mutation (Ser169del) in a Chinese Family With Early‐Onset Alzheimer's Disease,” Neuroscience Letters 468, no. 1 (2010): 34–37. 10.1016/j.neulet.2009.10.055 19853643

[28] G. Yang , R. Zhou , Q. Zhou , et al., “Structural Basis of Notch Recognition by Human γ‐Secretase,” Nature 565, no. 7738 (2019): 192–197. 10.1038/s41586-018-0813-8 30598546

[29] R. Zhou , G. Yang , X. Guo , Q. Zhou , J. Lei , and Y. Shi , “Recognition of the Amyloid Precursor Protein by Human γ‐Secretase,” Science (New York, NY) 363, no. 6428 (2019) eaaw0930. 10.1126/science.aaw0930 30630874

[30] R. F. Clark , M. Hutton , M. Fuldner , et al., “The Structure of the Presenilin 1 (S182) Gene and Identification of Six Novel Mutations in Early Onset AD Families,” Nature Genetics 11, no. 2 (1995): 219–222. 10.1038/ng1095-219 7550356

[31] M. Pagnon de la Vega , C. Näslund , and R. M. Brundin , “Mutation Analysis of Disease Causing Genes in Patients With Early Onset or Familial Forms of Alzheimer's Disease and Frontotemporal Dementia,” BMC Genomics [Electronic Resource] 23, no. 1 (2022): 1–8. 10.1186/s12864-022-08343-9 PMC8817590 35120450

[32] S. Hsu , A. A. Pimenova , K. Hayes , et al., “Systematic Validation of Variants of Unknown Significance in APP, PSEN1 and PSEN2,” Neurobiology of Disease 139 (2020): 104817–104817. 10.1016/j.nbd.2020.104817 PMC7236786 32087291

[33] N. Li , K. Liu , Y. Qiu , et al., “Effect of Presenilin Mutations on APP Cleavage; Insights Into the Pathogenesis of FAD,” Frontiers in Aging Neuroscience 8, no. MAR (2016): 51. 10.3389/fnagi.2016.00051 PMC4786568 27014058

[34] M. A. Gama Sosa , R. De Gasperi , A. B. Rocher , et al., “Age‐Related Vascular Pathology in Transgenic Mice Expressing Presenilin 1‐Associated Familial Alzheimer's Disease Mutations,” American Journal of Pathology 176, no. 1 (2010): 353–368. 10.2353/ajpath.2010.090482 PMC2797896 20008141

[35] Y. Wang , N. H. Greig , Y. Qs , and M. P. Mattson , “Presenilin‐1 Mutation Impairs Cholinergic Modulation of Synaptic Plasticity and Suppresses NMDA Currents in Hippocampus Slices,” Neurobiology of Aging 30, no. 7 (2009): 1061–1068. 10.1016/j.neurobiolaging.2007.10.009 PMC2717610 18068871

[36] B. I. Pérez‐Revuelta , A. Fukumori , S. Lammich , A. Yamasaki , C. Haass , and H. Steiner , “Requirement for Small Side Chain Residues Within the GxGD‐Motif of Presenilin for γ‐Secretase Substrate Cleavage,” Journal of Neurochemistry 112, no. 4 (2010): 940–950. 10.1111/j.1471-4159.2009.06510.x 20021564

[37] M. Cruts , H. Backhovens , S. Y. Wang , et al., “Molecular Genetic Analysis of Familial Early‐Onset Alzheimer's Disease Linked to Chromosome 14q24,” Human Molecular Genetics 4, no. 12 (1995): 2363–2371. 10.1093/hmg/4.12.2363 8634711

[38] L. Chávez‐Gutiérrez , L. Bammens , I. Benilova , et al., “The Mechanism of γ‐Secretase Dysfunction in Familial Alzheimer Disease,” EMBO Journal 31, no. 10 (2012): 2261–2261. 10.1038/emboj.2012.79 PMC3364747 22505025

[39] L. Sun , R. Zhou , G. Yang , and Y. Shi , “Analysis of 138 Pathogenic Mutations in Presenilin‐1 on the in Vitro Production of Aβ42 and Aβ40 Peptides by γ‐Secretase,” in Proceedings of the National Academy of Sciences of the United States of America (2017), E476–E485. 10.1073/pnas.1618657114 PMC5278480 27930341

[40] P. Poorkaj , V. Sharma , L. Anderson , et al., “Missense Mutations in the Chromosome 14 Familial Alzheimer's disease Presenilin 1 Gene,” Human Mutation 11, no. 3 (1998): 216–221. 10.1002/(SICI)1098-1004(1998)11:3<216::AID-HUMU6>3.0.CO;2-F 9521423

[41] E. A. Heilig , U. Gutti , T. Tai , J. Shen , and R. J. Kelleher , “Trans‐Dominant Negative Effects of Pathogenic PSEN1 Mutations on γ‐Secretase Activity and Aβ Production,” Journal of Neuroscience 33, no. 28 (2013): 11606–11617. 10.1523/JNEUROSCI.0954-13.2013 PMC3724549 23843529

[42] D. Campion , C. Dumanchin , D. Hannequin , et al., “Early‐Onset Autosomal Dominant Alzheimer Disease: Prevalence, Genetic Heterogeneity, and Mutation Spectrum,” American Journal of Human Genetics 65, no. 3 (1999): 664–670. 10.1086/302553 PMC1377972 10441572

[43] N. S. Ryan , J. M. Nicholas , P. S. J. Weston , et al., “Clinical Phenotype and Genetic Associations in Autosomal Dominant Familial Alzheimer's Disease: A Case Series,” Lancet Neurology 15, no. 13 (2016): 1326–1335. 10.1016/S1474-4422(16)30193-4 27777022

[44] D. Petit , S. G. Fernández , K. M. Zoltowska , et al., “Aβ Profiles Generated by Alzheimer's Disease Causing PSEN1 Variants Determine the Pathogenicity of the Mutation and Predict Age at Disease Onset,” Molecular Psychiatry 27, no. 6 (2022): 2821–2832. 10.1038/s41380-022-01518-6 PMC9156411 35365805

[45] H. Qing , W. Zhou , M. A. Christensen , X. Sun , Y. Tong , and W. Song , “Degradation of BACE by the Ubiquitin‐Proteasome Pathway,” Faseb Journal 18, no. 13 (2004): 1571–1573. 10.1096/fj.04-1994fje 15289451

[46] X. Sun , Y. Wang , H. Qing , et al., “Distinct Transcriptional Regulation and Function of the human BACE2 and BACE1 Genes,” Faseb Journal 19, no. 7 (May 2005): 739–749. 10.1096/fj.04-3426com 15857888

[47] K. Takahashi , T. Niidome , A. Akaike , T. Kihara , and H. Sugimoto , “Amyloid Precursor Protein Promotes Endoplasmic Reticulum Stress‐induced Cell Death via C/EBP Homologous Protein‐Mediated Pathway,” Journal of Neurochemistry 109, no. 5 (2009): 1324–1337. 10.1111/j.1471-4159.2009.06067.x 19476545

[48] L. Vaillant‐Beuchot , A. Mary , and R. Pardossi‐Piquard , “Accumulation of Amyloid Precursor Protein C‐Terminal Fragments Triggers Mitochondrial Structure, Function, and Mitophagy Defects in Alzheimer's Disease Models and Human Brains,” Acta Neuropathologica 141, no. 1 (2021): 39–65. 10.1007/s00401-020-02234-7 PMC7785558 33079262

[49] A. Jan , O. Gokce , R. Luthi‐Carter , and H. A. Lashuel , “The Ratio of Monomeric to Aggregated Forms of Abeta40 and Abeta42 Is an Important Determinant of Amyloid‐beta Aggregation, Fibrillogenesis, and Toxicity,” Journal of Biological Chemistry 283, no. 42 (2008): 28176–28189. 10.1074/jbc.M803159200 PMC2661389 18694930

[50] D. Xia , H. Watanabe , B. Wu , et al., “Presenilin‐1 Knockin Mice Reveal Loss‐of‐Function Mechanism for Familial Alzheimer's Disease,” Neuron 85, no. 5 (2015): 967–981. 10.1016/j.neuron.2015.02.010 PMC4358812 25741723

[51] N. Li , Y. Qiu , H. Wang , J. Zhao , and H. Qing , “PS1 Affects the Pathology of Alzheimer's Disease by Regulating BACE1 Distribution in the ER and BACE1 Maturation in the Golgi Apparatus,” International Journal of Molecular Sciences 23, no. 24 (2022): 16151. 10.3390/ijms232416151 PMC9782474 36555791

[52] L. Giliberto , R. Borghi , A. Piccini , et al., “Mutant Presenilin 1 Increases the Expression and Activity of BACE1,” Journal of Biological Chemistry 284, no. 14 (2009): 9027–9038. 10.1074/jbc.M805685200 PMC2666551 19196715

[53] C. Zhang , A. Browne , D. Y. Kim , and R. E. Tanzi , “Familial Alzheimer's Disease Mutations in Presenilin 1 Do Not Alter Levels of the Secreted Amyloid‐beta Protein Precursor Generated by Beta‐Secretase Cleavage,” Current Alzheimer Research 7, no. 1 (2010): 21–26. 10.2174/156720510790274428 PMC3260056 20205669

[54] M. Pera , D. Alcolea , R. Sánchez‐Valle , et al., “Distinct Patterns of APP Processing in the CNS in Autosomal‐Dominant and Sporadic Alzheimer Disease,” Acta Neuropathologica 125, no. 2 (2013): 201–213. 10.1007/s00401-012-1062-9 PMC3623032 23224319

[55] C. Hattori , M. Asai , Y. Oma , et al., “BACE1 Interacts with Nicastrin,” Biochemical and Biophysical Research Communications 293, no. 4 (2002): 1228–1232. 10.1016/S0006-291X(02)00351-0 12054507

[56] M. B. Podlisny , M. Citron , P. Amarante , et al., “Presenilin Proteins Undergo Heterogeneous Endoproteolysis Between Thr291 and Ala299 and Occur as Stable N‐ and C‐Terminal Fragments in Normal and Alzheimer Brain Tissue,” Neurobiology of Disease 3, no. 4 (1997): 325–337. 10.1006/nbdi.1997.0129 9173929

[57] K. Takeo , N. Watanabe , T. Tomita , and T. Iwatsubo , “Contribution of the γ‐Secretase Subunits to the Formation of Catalytic Pore of Presenilin 1 Protein,” Journal of Biological Chemistry 287, no. 31 (2012): 25834–25843. 10.1074/jbc.M111.336347 PMC3406669 22689582

[58] N. Willumsen , C. Arber , C. Lovejoy , et al., “The PSEN1 E280G Mutation Leads to Increased Amyloid‐β43 Production in Induced Pluripotent Stem Cell Neurons and Deposition in Brain Tissue,” Brain Communications 5, no. 1 (2023): fcac321. 10.1093/braincomms/fcac321 PMC9847549 36687397

[59] W. J. Luo , H. Wang , H. Li , et al., “PEN‐2 and APH‐1 Coordinately Regulate Proteolytic Processing of Presenilin 1,” Journal of Biological Chemistry 278, no. 10 (2003): 7850–7854. 10.1074/jbc.C200648200 12522139

[60] Y. Gu , F. Chen , N. Sanjo , et al., “APH‐1 Interacts With Mature and Immature Forms of Presenilins and Nicastrin and May Play a Role in Maturation of Presenilin.Nicastrin Complexes,” Journal of Biological Chemistry 278, no. 9 (2003): 7374–7380. 10.1074/jbc.M209499200 12471034

[61] S. H. Kim and S. S. Sisodia , “A Sequence Within the First Transmembrane Domain of PEN‐2 Is Critical for PEN‐2‐Mediated Endoproteolysis of Presenilin 1,” Journal of Biological Chemistry 280, no. 3 (2005): 1992–2001. 10.1074/jbc.M412404200 15537629

[62] G. Mao , M. Z. Cui , T. Li , Y. Jin , and X. Xu , “Pen‐2 Is Dispensable for Endoproteolysis of Presenilin 1, and Nicastrin‐Aph Subcomplex Is Important for both γ‐Secretase Assembly and Substrate Recruitment,” Journal of Neurochemistry 123, no. 5 (2012): 837–844. 10.1111/jnc.12016 PMC3509485 22973949

[63] K. Ahn , C. C. Shelton , Y. Tian , et al., “Activation and Intrinsic Gamma‐Secretase Activity of Presenilin 1,” PNAS 107, no. 50 (2010): 21435–21440. 10.1073/pnas.1013246107 PMC3003001 21115843

[64] N. Elad , B. De Strooper , S. Lismont , et al., “The Dynamic Conformational Landscape of Gamma‐Secretase,” Journal of Cell Science 128, no. 3 (2015): 589–598. 10.1242/jcs.164384 PMC4311135 25501811

[65] D. D. Shaye and I. Greenwald , “Endocytosis‐Mediated Downregulation of LIN‐12/Notch Upon Ras Activation in *Caenorhabditis elegans* ,” Nature 420, no. 6916 (2002): 686–690. 10.1038/nature01234 12478297

[66] B. De Strooper , W. Annaert , P. Cupers , et al., “A Presenilin‐1‐Dependent Gamma‐Secretase‐Like Protease Mediates Release of Notch Intracellular Domain,” Nature 398, no. 6727 (1999): 518–522. 10.1038/19083 10206645

[67] C. A. Hoogstraten , J. A. M. Smeitink , F. G. M. Russel , and T. J. J. Schirris , “Dissecting Drug‐Induced Cytotoxicity and Metabolic Dysfunction in Conditionally Immortalized Human Proximal Tubule Cells,” Frontiers in Toxicology 4 (2022): 842396. 10.3389/ftox.2022.842396 PMC8915871 35295229

[68] H. Kim , S. C. Yoon , T. Y. Lee , and D. Jeong , “Discriminative Cytotoxicity Assessment Based on Various Cellular Damages,” Toxicology Letters 184, no. 1 (2009): 13–17. 10.1016/j.toxlet.2008.10.006 18992794

[69] X. Sun , Y. Tong , H. Qing , C. H. Chen , and W. Song , “Increased BACE1 Maturation Contributes to the Pathogenesis of Alzheimer's Disease in Down Syndrome,” Faseb Journal 20, no. 9 (2006): 1361–1368. 10.1096/fj.05-5628com 16816111

[70] Q. Zheng , B. Song , G. Li , et al., “USP25 inhibition Ameliorates Alzheimer's Pathology Through the Regulation of APP Processing and Aβ Generation,” Journal of Clinical Investigation 132, no. 5 (2022): e152170. 10.1172/JCI152170 PMC8884900 35229730

[71] G. He , H. Qing , Y. Tong , F. Cai , S. Ishiura , and W. Song , “Degradation of Nicastrin Involves both Proteasome and Lysosome,” Journal of Neurochemistry 101, no. 4 (2007): 982–992. 10.1111/j.1471-4159.2007.04449.x 17326768

