# Supplementary material for: Presenilin 1 Mutations Converge on Aβ42/Aβ40 Elevation via Distinct Mechanisms
Source: MedComm (2020). 2026 Sep 30;7(10):e70978. doi: 10.1002/mco2.70978 (PMC13625817; doi:10.1002/mco2.70978)
Supplement: Supplementary file 1 [file MCO2-7-e70978-s001.docx]

Presenilin 1 mutations converge on Aβ42/Aβ40 elevation via distinct mechanisms

Isabel Bestard-Lorigados^1^, Keenan Sterling^1^, Liwei Li^2^, Zhehao Lin^2^, Zhanghui Wu^2^, Qinxin Zhu^2^, Wenhao Pan^2^, Fang Cai^3*^, Weihong Song^1,2,3*^

^1^Townsend Family Laboratories, Department of Psychiatry, Graduate Program in Neuroscience, The University of British Columbia, Vancouver, British Columbia, Canada

^2^ Center for Geriatric Medicine, Institute of Aging and The First Affiliated Hospital, Zhejiang Key Laboratory of Alzheimer’s Disease, Wenzhou Medical University, Wenzhou, Zhejiang, China.

^3^ Oujiang Laboratory (Zhejiang Lab for Regenerative Medicine, Vision and Brain Health), Wenzhou, Zhejiang, China.

* To whom correspondence should be addressed: Dr. Weihong Song, Email: [weihong@wmu.edu.cn](mailto:weihong@wmu.edu.cn), Tel: +86-577-86699670; Dr. Fang Cai, Email: [caifang@ojlab.ac.cn](mailto:caifang@ojlab.ac.cn), Tel: +86-18274814517

**Supplementary Figures**

**Figure S1. PS1 mutations do not alter APP full-length (APP-FL) levels across multiple cell lines.** 20E2, BAW, and 2EB2 cells were transfected with PS1_WT_, PS1_ΔS169_, PS1_M146V_, PS1_G384A_, PS1_C410Y_, or PS1_Y115H_. APP-FL levels were assessed by Western blot using the C20 antibody, with GAPDH used as a loading control. **(A)** Representative Western blots of APP-FL in 20E2 cells, *n* = 3. **(B)** Quantification of APP-FL levels in 20E2 cells normalized to PS1_WT_. No significant differences were observed among PS1 variants (*p* > 0.05). **(C)** Representative Western blots of APP-FL in BAW cells, *n* = 3. **(D)** Quantification of APP-FL levels in BAW cells showed no significant changes between PS1 mutants and PS1_WT_, *p* > 0.05. **(E)** Representative Western blots of APP-FL in 2EB2 cells, *n* = 3. **(F)** Quantification of APP-FL levels in 2EB2 cells showed no significant differences across all PS1 variants compared with PS1_WT_ (*p* > 0.05). Approximate molecular weights are indicated: GAPDH (~36 kDa) and APP-FL (~100 kDa). Data are shown as mean ± SEM. Statistical analyses were performed using one-way ANOVA.

**Figure S2. PS1 mutations do not alter ADAM10, ADAM17, or PS2 protein levels across multiple cell lines.** 20E2, BAW, and 2EB2 cells were transfected with PS1_WT_, PS1_ΔS169_, PS1_M146V_, PS1_G384A_, PS1_C410Y_, or PS1_Y115H_. Protein levels of ADAM10, ADAM17, and PS2 were assessed by Western blot, with GAPDH used as a loading control. **(A, C, E)** Representative Western blots of ADAM10, ADAM17, and PS2 in 20E2 cells, respectively, *n* = 3. **(B, D, F)** Quantification of ADAM10, ADAM17, and PS2 protein levels in 20E2 cells normalized to PS1_WT_. No significant differences were observed among PS1 variants (*p* > 0.05). **(G, I, K)** Representative Western blots of ADAM10, ADAM17, and PS2 in BAW cells, respectively, *n* = 3. **(H, J, L)** Quantification of ADAM10, ADAM17, and PS2 protein levels in BAW cells showed no significant differences between PS1 mutants and PS1_WT_ (*p* > 0.05). **(M, O, Q)** Representative Western blots of ADAM10, ADAM17, and PS2 in 2EB2 cells, respectively, *n* = 3. **(N, P, R)** Quantification of ADAM10, ADAM17, and PS2 levels in 2EB2 cells showed no significant differences across all PS1 mutations compared with PS1_WT_ (*p* > 0.05). Approximate molecular weights are indicated: GAPDH (~36 kDa), ADAM10 (~95 kDa), ADAM17 (~130 kDa), and PS2 (~22 kDa). Data are shown as mean ± SEM. Statistical analyses were performed using one-way ANOVA.

**Figure S3. PS1 mutations do not alter ADAM10 and ADAM17 in N2AdKO cells.** N2AdKO cells were co-transfected with C99 and either PS1_WT_, PS1_ΔS169_, PS1_M146V_, PS1_G384A_, PS1_C410Y_, or PS1_Y115H_. Protein levels of ADAM10 and ADAM17 were assessed by Western blot, with GAPDH used as a loading control. **(A, C)** Representative Western blots of ADAM10 and ADAM17, respectively, *n* = 3. **(B, D)** Quantification of ADAM10 and ADAM17 protein levels normalized to PS1_WT_. No significant differences were observed among PS1 variants (*p* > 0.05). Approximate molecular weights are indicated: GAPDH (~36 kDa), ADAM10 (~95 kDa), ADAM17 (~130 kDa). Data are shown as mean ± SEM. Statistical analyses were performed using one-way ANOVA.

**Figure S4. PS1 mutations impair Hes1 promoter activity, except ΔS169, and NICD expression rescues cell viability in Notch-impairing mutants.** **(A)** Hes1 promoter activity was assessed in N2AdKO cells, which were transfected with PS1_WT_ or PS1 mutants (PS1_ΔS169_, PS1_M146V_, PS1_G384A_, PS1_C410Y_, and PS1_Y115H_), *n* = 4. Cells were co-transfected with a Hes1 reporter and a membrane-bound Notch fragment (MV), which requires γ-secretase-mediated cleavage to generate the active Notch intracellular domain. PZ (vector, pcDNA4) + MV serves as the baseline control, and all results are normalized to this. PS1_WT_ significantly increased Hes1 activity compared to the vector control (PZ + MV), whereas all mutations, except PS1_ΔS169_, showed reduced Hes1 activity (*p* < 0.001), indicating impaired Notch signalling. In contrast, PS1_ΔS169_ did not significantly alter Hes1 activity compared to PS1_WT_ (*p* > 0.05), consistent with its classification as a Notch-sparing mutation. Expression of ICV, the γ-secretase cleavage product of Notch, restored Hes1 activity and served as a positive control. **(B)** N2AdKO cells were transfected with PS1_WT_, PS1_M146V_, or PS1_C410Y_, alone or in combination with the Notch intracellular domain plasmid (ICV), *n* = 8. Cell viability was assessed using the MTS assay and expressed as a percentage relative to PS1_WT_. PS1_M146V_ and PS1_C410Y_ significantly reduced cell viability compared to PS1_WT_. Co-transfection with ICV and these two PS1 mutations restored cell viability to levels comparable to PS1_WT_. Data are shown as mean ± SEM. Statistical analyses were performed using one-way ANOVA with Dunnett’s post hoc test. ****p* < 0.001, *****p* < 0.0001.

**Figure S5. PS1 full-length (PS1-FL) and its cleaved fragments across PS1 variants in multiple cell lines.** 20E2, BAW, and 2EB2 cells were transfected with PS1_WT_, PS1_ΔS169_, PS1_M146V_, PS1_G384A_, PS1_C410Y_, or PS1_Y115H_. PS1 expression was assessed by Western blot using antibodies against PS1-FL, PS1 C-terminal fragment (PS1-CTF), or PS1 N-terminal fragment (PS1-NTF), with GAPDH or β-actin used as loading controls. (**A)** Representative Western blots of PS1-FL and PS1-CTF in 20E2 cells. **(B)** Representative Western blots of PS1-FL and PS1-CTF in BAW cells. **(C)** Representative Western blots of PS1-FL and PS1-NTF in 2EB2 cells. Approximate molecular weights are indicated: GAPDH (~ 36 kDa), β-actin (~43 kDa), PS1-FL (~ 46 kDa), PS1-CTF (~18 kDa), and PS1-NTF (~28 kDa).

**Figure S6. Full Western blot images of PS1 full length (PS1-FL) and PS1-N-terminal fragment (PS1-NTF) in N2AdKO cells.** N2AdKO cells were transfected with PS1_WT_, PS1_ΔS169_, PS1_M146V_, PS1_G384A_, PS1_C410Y_, PS1_Y115H_, or empty vector control (negative control for PS1). **(A)** Full membrane image probed with anti-PS1 antibody (PS1N), detecting both PS1-FL and PS1-NTF. Molecular weight markers are indicated on the left. **(B)** Corresponding full membrane image probed with β-actin antibody as a control. Approximate molecular weights are indicated: β-actin (~43 kDa), PS1-FL (~46 kDa), and PS1-NTF (~28 kDa).

**Figure S7. Pearson correlation analysis between PS1 endoproteolytic rate and Aβ production, and PS1 endoproteolytic rate and C99 levels.** N2AdKO cells were co-transfected with C99 and PS1 variants, including PS1_WT_, PS1_ΔS169_, PS1_M146V_, PS1_G384A_, PS1_C410Y_, and PS1_Y115H_. Aβ levels were measured via ELISA, *n* = 3. C99 levels were measured via Western Blot using C20 antibody, *n* = 3. The endoproteolytic rate was calculated by dividing the PS1 N-terminal fragment (NTF) by the PS1 Full-length (FL), which were measured via Western Blot using an anti-PS1 antibody, *n* = 3. No significant correlations were observed between endoproteolytic rate and **(A)** Aβ40 levels, **(B)** Aβ42 levels, **(C)** the Aβ42/Aβ40 ratio or **(D)** C99 levels (*r* = 0.632, *R²* = 0.399; *r* = 0.603, *R²* = 0.363; *r* = 0.0186, *R²* = 0.0003; and *r* = 0.0378, *R²* = 0.0014, respectively; all *p* > 0.05). Statistical analyses were performed using Pearson’s correlation.
